# Efficiency, Specificity and Temperature Sensitivity of Cas9 and Cas12a RNPs for DNA-free Genome Editing in Plants

**DOI:** 10.3389/fgeed.2021.760820

**Published:** 2022-01-12

**Authors:** Raviraj Banakar, Mollie Schubert, Gavin Kurgan, Krishan Mohan Rai, Sarah F. Beaudoin, Michael A. Collingwood, Christopher A. Vakulskas, Kan Wang, Feng Zhang

**Affiliations:** ^1^ Department of Plant and Microbial Biology, University of Minnesota, St. Paul, MN, United States; ^2^ Center for Precision Plant Genomics, University of Minnesota, St. Paul, MN, United States; ^3^ Center for Genome Engineering, University of Minnesota, St. Paul, MN, United States; ^4^ Integrated DNA Technologies, Coralville, IA, United States; ^5^ Department of Agronomy, Iowa State University, Ames, IA, United States; ^6^ Crop Bioengineering Center, Iowa State University, Ames, IA, United States

**Keywords:** *Nicotiana benthamiana*, pennycress, protoplast, ribonucleoprotein, *Setaria viridis*, soybean, transfection, transformation

## Abstract

Delivery of genome editing reagents using CRISPR-Cas ribonucleoproteins (RNPs) transfection offers several advantages over plasmid DNA-based delivery methods, including reduced off-target editing effects, mitigation of random integration of non-native DNA fragments, independence of vector constructions, and less regulatory restrictions. Compared to the use in animal systems, RNP-mediated genome editing is still at the early development stage in plants. In this study, we established an efficient and simplified protoplast-based genome editing platform for CRISPR-Cas RNP delivery, and then evaluated the efficiency, specificity, and temperature sensitivity of six Cas9 and Cas12a proteins. Our results demonstrated that Cas9 and Cas12a RNP delivery resulted in genome editing frequencies (8.7–41.2%) at various temperature conditions, 22°C, 26°C, and 37°C, with no significant temperature sensitivity. LbCas12a often exhibited the highest activities, while AsCas12a demonstrated higher sequence specificity. The high activities of CRISPR-Cas RNPs at 22° and 26°C, the temperature preferred by plant transformation and tissue culture, led to high mutagenesis efficiencies (34.0–85.2%) in the protoplast-regenerated calli and plants with the heritable mutants recovered in the next generation. This RNP delivery approach was further extended to pennycress (*Thlaspi arvense*), soybean (*Glycine max*) and *Setaria viridis* with up to 70.2% mutagenesis frequency. Together, this study sheds light on the choice of RNP reagents to achieve efficient transgene-free genome editing in plants.

## Introduction

Clustered Regularly Interspaced Short Palindromic Repeats (CRISPR) and CRISPR-associated protein (Cas) systems were first discovered to cleave invading bacteriophage DNA as a prokaryotic adaptive immune system (Garneau et al., 2010; Horvath and Barrangou, 2010). Since then, CRISPR-Cas systems have been widely adopted to make precise sequence alterations in the genome of many species including plants (Chen et al., 2019). Among the diverse CRISPR-Cas systems, Cas9 and Cas12a are the most exploited for genome editing (Chen et al., 2019; Zhang et al., 2019; Zhang et al., 2021c). These systems typically consist of two components: the Cas nuclease protein and a CRISPR guide RNA (gRNA). The gRNA usually contains a 20–30 bp target-specific sequence along with a universal sequence that interacts with Cas protein to form an active ribonucleoprotein (RNP) complex. The RNP complex is directed to its target DNA sequence *via* RNA/DNA base pairing. RNA/DNA hybridization triggers double-stranded DNA breaks (DSBs) at the target site by the Cas nuclease to initiate the subsequent gene editing process (Chen et al., 2019).

Efficient genome editing requires effective delivery approaches to introduce the CRISPR-Cas reagents into cells. For most plant species, CRISPR-Cas and a targeting gRNA are delivered as plasmid DNA either by *Agrobacterium*- or biolistic-mediated transformation methods (Atkins and Voytas, 2020). With these approaches, the plasmids usually need to integrate into the genome, and the expressed Cas protein and CRISPR RNA assemble to form functional RNPs for the targeted gene modifications. Although the transgene sequences can be segregated out by breeding, these methods are time consuming and have additional weaknesses. First, agrobacterium mediated T-DNA or biolistic transformation often result in random insertions of transgenes, complex chromosomal rearrangements and other unintended genetic and epigenetic changes that are not easily rectified (Jupe et al., 2019; Liu et al., 2019). Second, the CRISPR-Cas constructs cannot always be segregated out, which could trigger regulatory and other public perception concerns (Jordan et al., 2017; Menz et al., 2020). For example, previous reports indicated that plasmid sequences could be inserted into DSB sites making them difficult to be segregated (Banakar et al., 2019; Dong et al., 2020). For plant species that have a lengthy juvenile growth period or are propagated vegetatively, the strategy of transgene-free segregants (null segregants) is also not pragmatic. Moreover, proper expression of the transgenic CRISPR-Cas cassette is essential to achieve efficient plant gene editing. It is not trivial to optimize the gene expression cassettes, including the promoters, terminators, and codon usage, for a new plant species (Chen et al., 2019). In addition, the expression of the CRISPR-Cas cassettes can be affected by the position of their integration resulting in transgene silencing (Fagard and Vaucheret, 2000). On the other hand, when the integrated cassettes are highly expressed, prolonged expression could lead to the increasing possibility of off-target mutations, and chimeric mutants (Chen et al., 2019).

To overcome these challenges, several strategies have been developed to achieve DNA-free gene editing in plants. This includes delivery of *in vitro* transcribed Cas mRNAs or pre-assembled CRISPR-Cas RNPs. While low genome editing efficiency was observed with RNA delivery in previous research (Zhang et al., 2016), RNP delivery of Cas9/Cas12a has resulted in efficient editing in a number of plants, including lettuce (*Lactuca sativa*), Arabidopsis, grape (*Vitis vinifera*), apple, *Petunia hybrida*, potato, tobacco, soybean, rice, maize and wheat (Woo et al., 2015; Malnoy et al., 2016; Subburaj et al., 2016; Svitashev et al., 2016; Andersson et al., 2018). In some examples, genome editing efficiencies using RNP delivery are equivalent to plasmid-based expression systems (Zhang et al., 2021b). This process offers not only technology advantages, such as bypassing the need of vector construction, but also less regulatory restriction (Chen et al., 2019). Because no recombinant DNA is transformed, plants edited by RNPs can be considered transgene free. In addition, since RNPs are only transiently present in plant cells after transformation, off-target effects and chimeric mutants caused by prolonged exposure to CRISPR-Cas systems are also minimized (Zhang et al., 2016; Kim et al., 2017).

Despite the great potential of CRISPR-Cas RNP systems, systematic studies by directly comparing their efficacies are lacking to provide guidelines for optimal selection of RNP reagents. In a recent study, Banakar et al. compared three SpCas9 variants, wildtype SpCas9 (Cas9_WT), high fidelity SpCas9 (Cas9_HiFi), SpCas9 D10A nickase, and two wildtype Cas12a nucleases (AsCas12a and LbCas12a) in rice calli. As a result, LbCas12a exhibited a higher editing efficiency than that of Cas9_WT, Cas9_HiFi and AsCas12a with the notable temperature sensitivity (Banakar et al., 2020). In addition, when delivered as DNA plasmids into plants, the Cas9 and Cas12a proteins have shown reduced nuclease activities at lower temperature (22–28°C) than the elevated temperature (37–42°C) (LeBlanc et al., 2018; Malzahn et al., 2019; Schindele and Puchta, 2020). Because most plant tissue culture and transformation require the low temperature condition, it will be important to thoroughly evaluate the temperature sensitivity of the CRISPR-Cas systems when used as RNPs. In this work, we established an efficient and simplified protoplast-based genome editing platform for CRISPR-Cas RNP delivery in multiple plant species. By testing six commercially available Cas9 and Cas12a variants, Cas9_WT, Cas9_HiFi, AsCas12a_WT, AsCas12a_Ultra, LbCas12a_WT and LbCas12a_V4 in *Nicotiana benthamiana*, we sought to evaluate the efficiency, specificity and temperature sensitivity of the widely available CRISPR-Cas enzymes delivered as RNP systems. Additionally, we demonstrated RNP mediated editing in pennycress (*Thlaspi arvense*), soybean (*Glycine max*) and *Setaria viridis* protoplasts. Our work indicated that RNP-mediated gene editing in plant protoplasts is a promising technique for quickly screening and optimizing the CRISPR-Cas systems as well as an effective platform for generating transgene-free gene edited plants.

## Materials and Methods

### Plant Growth Conditions

For *Nicotiana benthamiana*, plant growth protocol was followed as described by Li et al. (2016). In short, seeds of the GFP16c line (Ruiz et al., 1998) were first sterilized in 70% ethanol for 2 min, then in 2.5% Sodium hypochlorite for 10 min, followed by three rounds of wash with sterile water. Twenty to thirty sterilized seeds were sown on growth media plates for germination at 25 ± 2°C under 16/8 h of light and dark. After 15 days, the healthy seedlings were transferred to medium sized pots to grow for a month period in growth chamber (16/8 h) with 22/20°C of day/night temperature.

For Pennycress, seed germination and plant growth were conducted in growth chamber (16/8 h) with 22°C/20°C of day/night temperature. After 15 days of germination, young leaves were collected for protoplast isolation and transfection.

For *Setaria viridis*, seeds of *Setaria viridis* cv. ME34 were germinated and grown in growth chambers with the settings: temperature 31°C/22°C (day/night), photoperiod 16/8 h (day/night), relative humidity 30% and light intensity ∼250–400 μE/m^2^/s. Young leaves from 12–15 days old plants were collected for protoplast isolation and transformation.

For soybean, seeds of soybean cultivar Williams 82 was grown in greenhouse till flowering. Immature seed pods at the R2 stage were harvested for protoplast isolation and transfection using the same protocol as described for *Nicotiana benthamiana.*

### Protoplast Isolation and Transfection

*Nicotiana benthamiana* protoplasts were isolated from the second and third expanding leaf from the top using the simplified protocol based on that developed earlier (Li et al., 2016). Briefly, leaves, sterilized with 0.5% bleach for 5 min, were cut into small pieces of 0.2 mm using a sharp scalpel and blade, and transferred to digestion media (DM, 0.45 M mannitol, 5 mM MES, 0.8% cellulase R10, and 0.2% Macerozyme R10) for 4–6 h. Digested leaf sections were filtered through 100 µM filter to 50-ml falcon tube and centrifuged at 100 xg for 5 min to collect protoplasts. After suspended in 5 ml of the washing buffer (WB, 0.45 mM mannitol, and 10 mM CaCl_2_), the protoplast-containing solution was mixed with 10 ml of 0.55 M sucrose and centrifuged for 10 min at 100 xg to separate intact protoplasts from debris. The protoplasts floating at the interphase layer were carefully transferred into a 50-ml falcon tube containing 5 ml of WB. The protoplasts were washed three times by repeating the cycle of centrifuging at 100 xg for 5 min. Each time, the supernatant was discarded, and the protoplasts were resuspended in 5 ml WB. At the final step, protoplasts were resuspended in appropriate volume (∼1 ml) of MMG buffer (0.4 M Mannitol, 4 mM MES, pH 5.7, and 15 mM MgCl_2_) to reach a density of 1 × 10^6^ cell/mL.

In each transfection, 200 µL of protoplast suspension was mixed with the RNP complex solution in the transfection media (TM, 0.2 M mannitol, 100 mM CaCl_2_, and 40% PEG-4000). The transfection mixture was incubated for 15 min in the dark followed by the twice washing cycle as described above. At the final washing step, protoplasts were resuspended in 2 ml of protoplast culture nutrient solution (PCN, 0.4 M mannitol, 4.3 g/L MS powder with B5 vitamins, 15 g/L sucrose, 0.1 mg/L 6-benzylaminopurine (BAP) and 1-naphthaleneacetic acid (NAA), pH 5.7), transferred into a 12 well plates and placed in dark for 48 h at 22, 26 or 37°C.

For soybean, seeds of soybean cultivar Williams 82 was grown in greenhouse till flowering. Immature seed pods at the R2 stage were harvested. Pods were sterilized for 2 min in 70% ethanol, 10 min in 0.5% bleach followed by three times of wash in sterile water. Immature seeds were removed from pods, cut into 1 mm pieces, and placed into digestion media for overnight digestion (12–16 h)**.** Protoplast isolation and transformation in pennycress, soybean and *Setaria viridis* were performed using the same protocol as described above for *Nicotiana benthamiana.* A total of three technical protoplast transformation replicates were carried out over a period of 3 days for each guide RNA/protein combination.

### Protoplast Regeneration and Tissue Culture

Regeneration and tissue culture of *N. benthamiana* protoplasts were performed following the protocol developed earlier (Li et al., 2016). Briefly, the transformed protoplasts from *N. benthamiana* GFP16c line in the PCN media were incubated under the condition of 16/8 h light/dark at 26°C. Once the calli reached 0.1–0.2 mm in size (normally after 17–20 days), they are transferred onto the solid protoplast culture media (PCM, 4.3 g/L MS powder with B5 vitamins, 17 g/L sucrose, 1 mg/L BAP, 0.1 mg/L NAA, 7 g/L Agar, pH 5.7) in the growth incubator under the condition of 16/8 h light/dark at 26°C. After 2 weeks of calli expansion, the sizable calli were subcultured 2–3 times every 2 weeks using the same media until shoots were well developed. Healthy shoots are transferred to the rooting media (RM, 4.3 g/L MS powder with B5 vitamins, 10 g/L sucrose, 0.2 mg/L NAA, 7 g/L Agar, pH 5.7) for 2–3 weeks and can be transferred to soil for seeds.

### CRISPR Guide RNA Design and RNP Assembly

In *N. benthamiana*, the CRISPR target sites were manually identified by scanning the GFP and *PDS* coding sequences. The Cas9 and Cas12a guide RNA targeting sites were overlapping to allow direct comparison of their editing efficiencies. In pennycress, *Setaria viridis* and soybean, the CRISPR/Cas9 targeting sites were chosen based in the previous reports (Supplementary Table S1), while the CRISPR/Cas12a targeting sites were manually identified for sequences close to the Cas9 sites.

All the CRISPR RNAs were synthesized at Integrated DNA Technologies Inc. (IDT, Coralville, IA, United States) as crRNA molecules (2 nmol) along with tracrRNA (5 nmol) or as 2 nmol single guide RNA (sgRNA). The crRNA and tracrRNA or sgRNA were dissolved in 20–50 μL of nuclease free-IDTE buffer (1 × TE buffer, pH 7.5) to a concentration of 100 μM of each. Equal molar concentration of crRNA and tracrRNA were mixed and incubated at 95°C for 5 min followed by room temperature (22°C) for 10 min. To assemble the RNP *in vitro*, 200 pmol of guide RNA molecules were mixed with 20 μg (2 μL) of the Cas proteins, made by the IDT team, along with 2 μL of 1 × PBS buffer (pH 7.4). After 10 min of incubation at room temperature, this RNP complex will be ready for protoplast delivery. All the Cas proteins used in this study can be purchased or requested through IDT directly. The catalog numbers are 1081058, 1081060, 1081068 and 10001272 for SpCas9_WT, SpCas9_HiFi, AsCas12a_WT and AsCas12a_Ultra, respectively. The LbCas12a_WT and V4 versions were R&D stock and described in the recent study that can be available as a custom order upon request (Dong et al., 2021).

### Mutation Detection and Next Generation Sequencing Analysis

The protocol for DNA extraction from protoplasts and regenerated plants was described previously (Weiss et al., 2020). DNA from each technical replicate was independently isolated and used for PCR amplification. The genomic region surrounding each target site was amplified using the rhAmpSeq CRISPR Library Kit (Integrated DNA Technologies, Coralville, IA, United States) with 50 ng of template DNA, forward and reverse primers (Supplementary Table S2). The primers contained “tails” to add sample-unique P5 and P7 indexes for Illumina sequencing in two rounds of PCR. PCR amplicons were sequenced on an Illumina MiSeq instrument (v2 chemistry, 150 bp paired end reads) (Illumina, San Diego, CA, United States). Resulting NGS data was analyzed for genome editing with CRISPAltRations v1.0 using default parameters for window sizes detecting Cas9 (8 bp) or Cas12a (9 bp) editing (Kurgan et al., 2021). At each target site, mutation efficiency was calculated as the percentage of total reads containing an indel within the specified window around the cut site. Average of three technical replicates was derived for each target site and presented as editing efficiency. The NGS data were deposited into the Sequence Read Archive (SRA) hosted by NCBI with the accession number PRJNA745954.

### Mutant Characterization in E1 Plants

The seeds harvested from the candidate E0 plants were germinated and grown on the growth media plate (Li et al., 2016). After 2 weeks of germination, the plantlets were examined under fluorescence stereomicroscope (Leica MZ FL III, Leica Microscopy Systems Ltd., Switzerland). Genomic DNA were extracted from candidate the GFP negative or reduced plants to perform PCR amplification in the targeted sequences. To distinguish individual mutations from mono-allelic or bi-allelic mutant plants, the PCR amplicons from each plant sample were cloned into the pJET1.2 vector using the CloneJET PCR Cloning Kit (Thermo scientific Inc., MA, U.S.A.). Twelve clones from each sample were subjected to Sanger sequencing to identify mutations.

### Statistical Analysis

A paired *t*-test was used to compare the mutation frequencies of RNP-transfected protoplast samples under different conditions.

## Results

### Cas9 and Cas12a RNPs Induce High Targeted Mutagenesis Efficiencies in *N. benthamiana* Protoplasts

**FIGURE 1:**
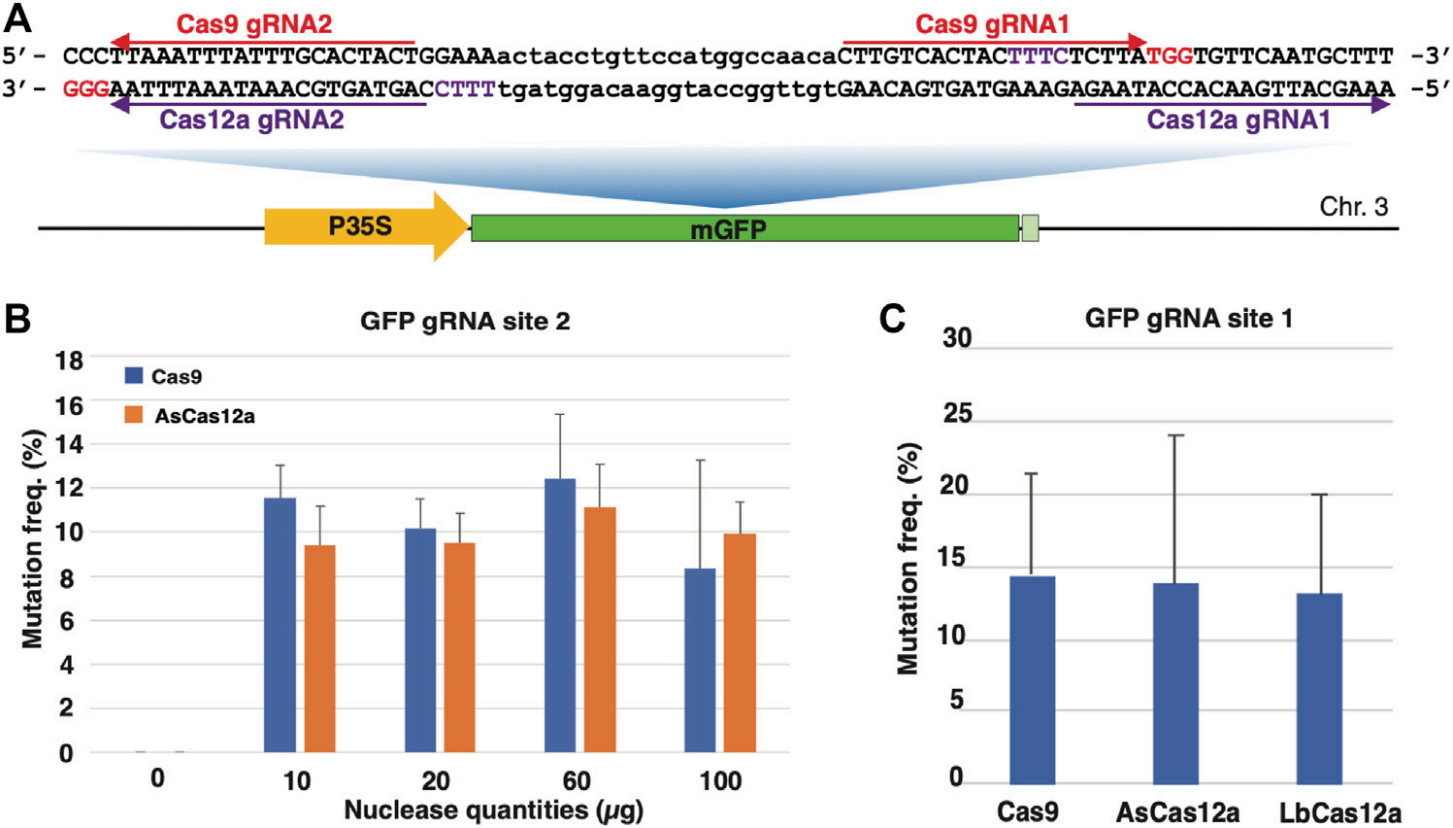
CRISPR/Cas RNP delivery in protoplasts from *N. benthamiana* GFP16c line. **(A)** The schematic illustration of the single-copy mGFP gene and the gRNA target sites in the *N. benthamiana* GFP16c line. The Cas9 gRNA targeted sequences are indicated by the red arrow lines with PAM sequences highlighted in red. The Cas12a gRNA targeted sequences are indicated by the purple arrow lines with the PAM sequences highlighted in purple. The mGFP gene is located on chromosome 3 with the coding sequence represented by the green box, the CaMV 35S promoter region represented by the yellow box and the terminator region represented by the light green box. **(B)** Optimization of the RNP delivery with different quantities of the Cas9 protein. The *X* axis represents the Cas9 RNP quantities. The *Y* axis indicates the targeted mutation frequencies. **(C)** The Cas9 and Cas12a RNPs induce efficient mutagenesis in *N. benthamiana* protoplasts. The *X* axis indicates the types of Cas RNPs. The *Y* axis indicates the targeted mutation frequencies. The error bars represent the standard deviations from 3 replicates in each RNP transfection experiment. The transfections with gRNA and nuclease alone were conducted as negative controls. No editing above sequencing background noise was found in the negative controls.

We first sought to establish the CRISPR-Cas RNP delivery system by transforming *Nicotiana benthamiana* protoplasts. In this study, a single-copy green fluorescent protein (GFP) transgenic *N. benthamiana* line, GFP16c (Ruiz et al., 1998), was used for protoplast isolation and polyethylene glycol (PEG)-mediated transformation using a simplified protocol from previous research (Zhang et al., 2013; Li et al., 2016). The RNP transfection and mutagenesis assessment procedure was outlined in Supplementary Figure S1. We selected four CRISPR targeted sites, two for each Cas9 and Cas12a, in the GFP coding sequence (Figure 1A). The CRISPR guide RNA sequences were chemically synthesized and assembled with the corresponding Cas proteins to form RNPs *in vitro*. Each RNP with a single CRISPR guide RNA was transfected and assessed individually in protoplasts.

To determine the optimal quantity of RNPs for mutagenesis, SpCas9 WT and AsCas12a WT RNPs with four different quantities, i.e. 10, 20, 60 and 100 μg, were first used to transfect GFP16c protoplasts. The guide RNAs used in each RNP were made to target the gRNA site two in the GFP coding sequence because of the identical target sequence for both types of nucleases (Figures 1A,B). Forty-eight hours after the transfection that was carried out at 22°C, the protoplast samples were collected and subjected to the mutagenesis assessment assay. The targeted region was amplified from the genomic DNA of each protoplast sample. The PCR amplicons were then sequenced using Next Generation Sequencing (NGS). The NGS reads from each sample, averaging 31,926 reads per sample, were analyzed to estimate the indel mutation frequency in the targeted sites. Figure 1B shows that similar levels of mutagenesis frequency were achieved with different RNP quantities. Mutagenesis frequencies of 11.6 ± 1.5%, 10.3 ± 1.3%, 12.4 ± 2.4% and 8.3 ± 4.9% were observed from protoplasts transfected with 10, 20, 60 and 100 µg of the SpCas9 WT RNP. Similar mutagenesis frequencies, 9.43 ± 1.77%, 9.52 ± 1.34%, 11.13 ± 1.97% and 9.92 ± 1.47%, were obtained from the samples transfected with 10, 20, 60 and 100 μg, respectively, of the AsCas12a WT RNP (Figure 1B). No statistically significant difference was observed between different quantities of each RNP. This suggested that RNP quantities tested in this study was not limiting genome editing efficacy. Thus, 20 µg CRISPR/Cas RNPs was used for protoplast transfection in the subsequent experiments.

Next, we compared the efficacy of wild type SpCas9 RNP with two widely used Cas12a nucleases, the wild type AsCas12a and LbCas12a, by targeting the overlapping Cas12a gRNA site 1 (Figure 1A). All three Cas RNPs demonstrated comparable mutagenesis efficiencies at 14.4 ± 7.1%, 13.7 ± 10.2% and 13.1 ± 6.8%, respectively (Figure 1C). Thus, using the PEG-mediated transfection approach, we were able to achieve efficient mutagenesis with both SpCas9 and Cas12a RNPs in *N. benthamiana* protoplasts.

### Assessing Temperature Sensitivity of Cas9 and Cas12a RNPs

To determine the temperature sensitivity of these CRISPR/Cas RNPs, we assessed genome editing under three temperature regimes, 22°, 26° and 37°C. In addition to the wild type SpCas9, AsCas12a, and LbCas12a, we also included three commercially available Cas9 and Cas12a variants, the high fidelity SpCas9 (Cas9 HiFi), an AsCas12a mutant (AsCas12a Ultra) and an LbCas12a mutant (LbCas12a V4), which demonstrated enhanced efficacy in human cell lines (Vakulskas et al., 2018; Zhang L. et al., 2021). To facilitate the direct comparisons between the Cas9 and Cas12a RNPs, the gRNA site 2 with an identical CRISPR target sequence for both types of nucleases was used for the assay (Figure 1A). Forty-8 hours after the transfection, the mutagenesis frequency was assessed in each sample using the NGS assay described above (Supplementary Figure S1).

**FIGURE 2:**
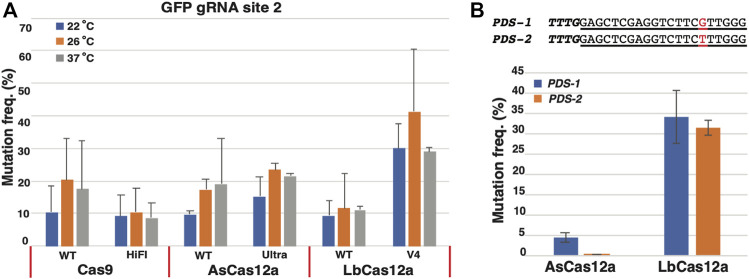
Comparison of temperature sensitivity and nuclease specificity of CRISPR/Cas RNPs in *N. benthamiana* protoplasts. **(A)** Assessment of temperature sensitivity for the Cas RNPs. Mutation frequencies (*Y* axis) induced by various Cas9 and Cas12a RNPs (*X* axis) under three different temperatures, 22°C (blue), 26°C (orange) and 37°C (grey), respectively. **(B)** Assessment of sequence specificity for AsCas12a and LbCas12a RNPs. The *PDS*1 and *PDS*2 targeted sequences is shown with the 1 bp mismatch highlighted in red. Mutation frequencies (*Y* axis) induced by AsCas12a_Ultra and LbCas12a_V4 RNPs (*X* axis) is indicated for each target site with blue for *PDS*1 and orange for *PDS*2. The error bars represent the standard deviations from 3 replicates in each RNP transfection experiment. A paired *t*-test was conduct between each temperature treatment for each CRISPR RNP. No statistic significance was observed between any samples. The transfections with gRNA and nuclease alone were conducted as negative controls. No editing above sequencing background noise was found in the negative controls.

We first examined the mutagenesis frequencies of each Cas protein. Efficient mutagenesis rates were observed for all Cas RNPs at each temperature condition, ranging from 9.2 to 30.0% at 22°C, 10.5–41.2% at 26°C and 8.7–28.9% at 37°C (Figure 2A). Next, we compared the genome editing efficiencies between the wild types and their derivative variants. As shown in Figure 2A, Cas 9 WT appeared to outperform Cas 9 HiFi at 26 and 37°C. On the contrary, in the Cas12a family, AsCas12a Ultra and LbCas12a V4 exhibited comparable or higher activities than their wild type parents at all temperature conditions. Notably, compared to other tested Cas RNPs, LbCas12a V4 demonstrated the highest mutagenesis rates at 30.0, 41.2 and 28.9% from 22°C to 37°C, respectively (Figure 2A).

When the temperature sensitivity patterns were examined for each Cas RNP, they can be generally classified into two categories. In the first category, the modest but not statistically significant increases in the mutagenesis frequencies were observed from Cas9 WT, AsCas12a WT, AsCas12a Ultra and LbCas12a V4 in response to the elevated temperatures from 22°C to 26°C. As such, 2.0-, 1.8-, 1.5- and 1.4-fold increases in mutagenesis rates were found from Cas9 WT (10.3–20.3%), AsCas12a WT (9.7–17.4%), AsCas12a Ultra (15.3–23.5%) and LbCas12a V4 (30.0–41.2%), respectively (Figure 2A). When the temperature was further elevated from 26°C to 37°C, however, no additional improvement was observed in the nuclease activities of these Cas RNPs. Their mutagenesis frequencies either stayed at the similar level as found in Cas9 WT (20.3 versus 17.6%), AsCas12a WT (17.4 versus 19.1%) and AsCas12a Ultra (23.5 versus 21.4%), or decreased from 41.2 to 28.9% in LbCas12a V4 (Figure 2A). By contrast, in the second category, no significant changes in mutagenesis frequencies were observed from Cas9 HiFi (9.2, 10.5 and 8.7%) and LbCas12a WT (9.4, 11.6 and 11.1%) under all the temperature conditions (Figure 2A). Taken together, all SpCas9, AsCas12a and LbCas12a RNPs tested in this study demonstrated efficient mutagenesis activities in protoplasts under three temperature regimes; no statistically significant temperature sensitivity was observed for each Cas protein.

### Evaluating Sequence Specificity of AsCas12a and LbCas12a RNPs

Previous studies in human cells suggested that LbCas12a possessed higher nuclease activity but lower sequence specificity than AsCas12a (Kleinstiver et al., 2016). Yet no direct comparisons of these Cas12a proteins were reported in plants (Malzahn et al., 2019). To investigate their specificity in plant cells, we selected a nearly identical Cas12a target site with only 1 bp mismatch at position 15 from the *N. benthamiana* phytoene desaturase (*PDS*) homologous genes *PDS*-1 and *PDS*-2 (Figure 2B). The CRISPR guide RNA was designed, synthesized and assembled with the AsCas12a Ultra and LbCas12a V4 proteins to target the *PDS*-1 site. The respective RNPs were transformed into GFP16c protoplasts and assessed for the mutagenesis frequency at 22°C using the same procedure described above. The PCR amplicons were obtained from both *PDS-1* and *PDS-2* sequences using the conserved primer pair. The mutation rate was estimated in each amplicon sample using the NGS assay. In each assay, the NGS reads were separated into the individual *PDS*-1 and *PDS*-2 sequences using the single nucleotide polymorphisms (SNPs, Supplementary Figure S2).

As shown in Figure 2B, LbCas12a V4 RNP induced the similar mutation frequencies in both target sites, with 34.0 ± 6.4% on *PDS-1* and 31.3 ± 1.9% on *PDS-2*. In contrast, the AsCas12a Ultra RNP displayed a strong discrimination between two target sites with the differing mutation rates by 84 folds, with 4.2 ± 1.2% on allele 1 and 0.05 ± 0.05% on allele 2 (Figure 2B). Thus, the LbCas12a V4 RNP induced higher mutagenesis efficiency than the AsCas12a Ultra RNP, but AsCas12a Ultra offered greater sequence specificity for the investigated mismatch position.

### Efficient and Heritable Mutagenesis Induced by CRISPR-Cas RNPs in Regenerated Calli and Plants

**FIGURE 3:**
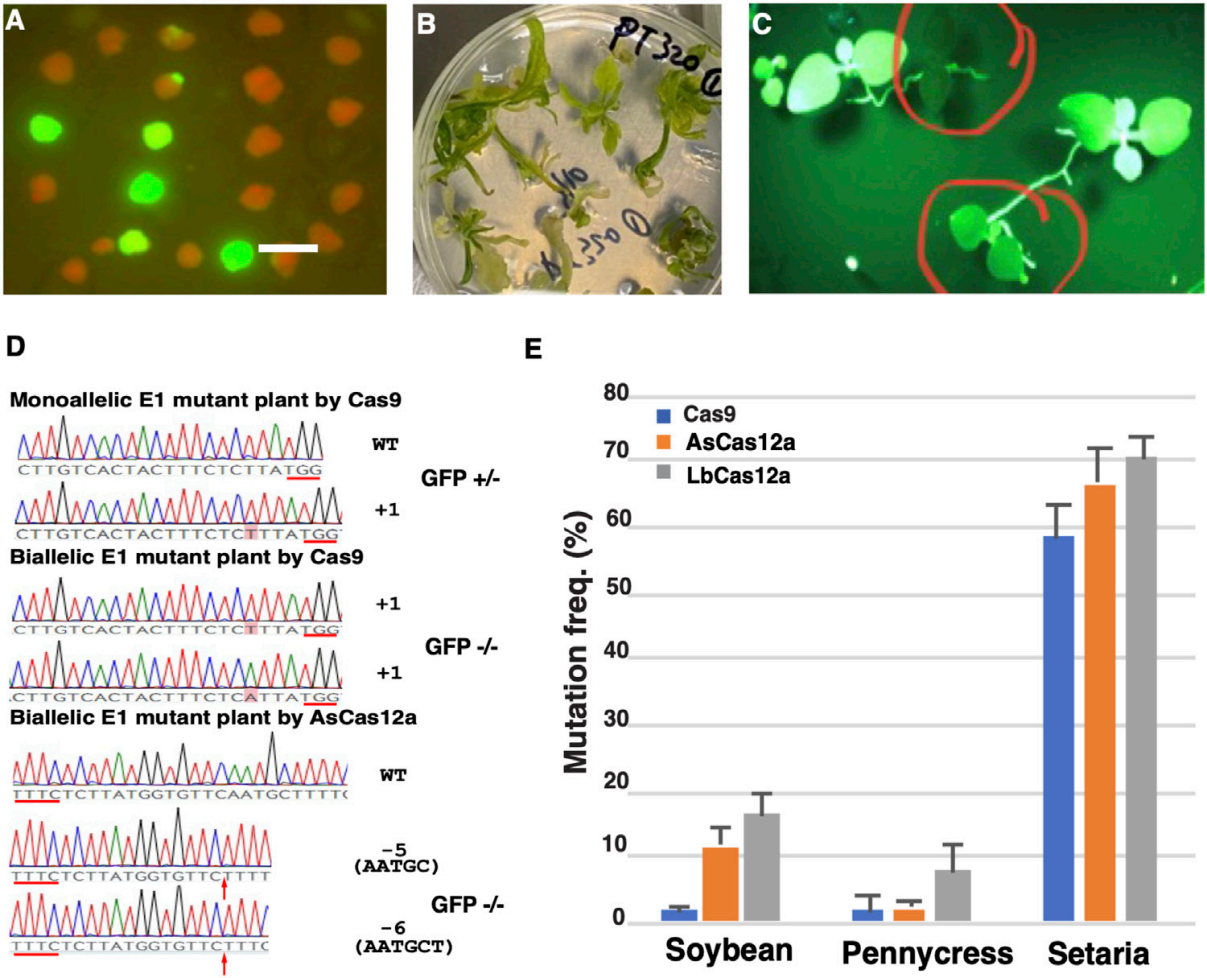
Heritable mutations induced by CRISPR/Cas RNPs in the regenerated E0 calli and E1 plants. **(A)** GFP negative calli with the high frequency visualized under GFP channel. Scale bar: 1 cm. **(B)** E0 plantlets regenerated from RNP transfected protoplasts. **(C)** GFP knock-out plants in E1 progeny. Bi-allelic or mono-allelic mutant plants are indicated in the red circles with either complete loss or reduced intensity of green fluorescence, respectively. **(D)** Mutation profiles of the E1 plants with GFP negative and semi-negative phenotypes. Insertion or deletion mutations in the targeted sequences are shown in the chromatogram snapshots from Sanger sequencing. The nucleotides from insertions are shaded in red. The break point of each deletion is indicated by the red arrow with the deleted nucleotides shown in parentheses. The PAM sequences of each targeted site are underlined with the red lines. The corresponding phenotype for each E1 plant is shown as either GFP negative (GFP −/−) or semi-negative (GFP +/-). **(E)** Cas 9 and Cas12a RNPs induced efficient mutagenesis in the protoplasts from diverse plant species. Mutation frequencies (*Y* axis) induced by SpCas9_WT (blue), AsCas12a_Ultra (orange) and LbCas12a_V4 RNPs (grey) are shown in soybean, pennycress and *S. viridis* (*X* axis). The error bars represent the standard deviations from 3 replicates in each RNP transfection experiment. The transfections with gRNA and nuclease alone were conducted as negative controls. No editing above sequencing background noise was found in the negative controls.

We next evaluated the mutagenesis efficiencies of CRISPR-Cas RNPs in regenerated calli and plants. The Cas9 WT, Cas9 HiFi, AsCas12a Ultra and LbCas12a V4 RNPs targeting the gRNA site one in the GFP coding sequence were transformed into GFP16c protoplasts under 22°C (Table 1). Using the regeneration protocol established previously in our group (Li et al., 2016), we obtained regenerated calli from all the transformation groups after about 4 weeks of transformation (except that only two calli were regenerated in the LbCas12a V4 group) (Figure 3A). In each group, the mutagenesis frequency was estimated by using the number of the calli with GFP negative sectors divided by the total number of calli (Figure 3A). As summarized in Table 1, the average mutagenesis frequencies were 55.8, 34 and 85.2% from Cas9 WT, Cas9 HiFi and AsCas12a Ultra transformed calli, respectively. The GFP negative sector-containing calli in each transformation group were also sampled for NGS analyses to confirm the occurrence of targeted indel mutations.

**TABLE 1:** Assessment of mutagenesis frequencies in regenerated calli (E0).

nuclease	Replicates	Total calli	# of GFP negative calli	% GFP negative calli	Average %^1^
Cas9 WT	1	10	7	70.0%	55.8%
	2	12	5	41.7%	
Cas9 HiFi	1	49	17	34.7%	34.0%
	2	9	3	33.3%	
AsCas12a Ultra	1	26	22	84.6%	85.2%
	2	28	24	85.7%	
LbCas12a V4	1	2	1	50%	N.A.^2^
	2	0	0	N.A.^2^	

Notes: 1. The average percentage of the GFP negative calli was estimated by taking the average of two transfection replications. 2. Transformation of LbCas12a V4 RNP yielded a low number of regenerated calli. The average percentage of GFP negative calli were not calculated because of the limited sample size.

To test the heritability of the targeted mutations, the mutation-containing calli in the Cas9 WT and AsCas12a Ultra groups were regenerated into plants (Figure 3B). Edited seeds (E1) were collected from three primary RNP transformed (E0) plants, two from Cas9 WT and one from AsCas12a Ultra edited E0 plants, respectively. When twenty seeds from each E0 plant were grown on the growth media, segregation of the GFP positive and negative mutant plants was clearly observed in each E1 population (Figure 3C). Plants with reduced GFP fluorescence were also observed in each population suggesting hemizygosity of the functional GFP gene. From each E1 population, five plants with either GFP negative or semi-negative phenotypes were genotyped to identify the mutation profile. Bi-allelic and mono-allelic mutations were confirmed in the GFP negative and semi-negative plants using sanger sequencing, respectively (Figure 3D and Supplementary Table S3).

### CRISPR-Cas RNPs Induced Efficient Mutagenesis in Diverse Plants

Next, we extended this CRISPR/Cas RNP transfection method to a diverse group of dicot and monocot plants, including pennycress (*Thlaspi arvense*), soybean (*Glycine max*) and *Setaria viridis* (Supplementary Figure S3). The SpCas9 WT, AsCas12a Ultra and LbCas12a V4 RNPs were introduced into the protoplasts of each species targeting a set of sequences listed in Supplementary Table S1 with mutagenesis efficiency assessed using NGS. As a result, Lb Cas12a V4 RNPs displayed the highest mutation efficiencies of 16.5 ± 3.4% in soybean, 8.0 ± 4.4% in pennycress and 70.2 ± 3.5% in *S. viridis*. AsCas12a Ultra RNPs yielded slightly lower mutation efficiencies with 11.7 ± 2.6%, 2.4 ± 0.6%, and 66.4 ± 5.5%, in soybean, pennycress and *Setaria*, respectively. In comparison, the SpCas9 WT RNPs showed the lowest mutagenesis efficiencies of 2.1 ± 0.7%, 3.0 ± 2.4% and 58.4 ± 5.1%, in soybean, pennycress and *Setaria*, respectively (Figure 3E). This result was consistent with our observations in *N. benthamiana* protoplasts confirming that the Cas12a RNPs, particularly LbCas12a WT and V4, have the greater potential to induce high mutagenesis in plants. Notably, all the CRISPR-Cas RNPs exhibited high mutation rates in *S. viridis* suggesting that its protoplast system is highly amenable for the CRISPR-Cas RNP mediated genome editing.

## Discussion

Although the use of CRISPR-Cas RNPs has been demonstrated in plant species, no systematic studies were conducted to provide the guideline for optimal selection of RNP reagents (Zhang et al., 2021b). In this study, after optimizing the PEG-mediated RNP delivery procedure, we sought to evaluate and compare the efficiency, specificity and temperature sensitivity of the three most widely used CRISPR-Cas system delivered as RNPs: SpCas9, AsCas12a and LbCas12a. One challenge in RNPs comparison is that it is difficult to measure its transfection frequency. A fluorescent protein, such as GFP, is typically used to visualize and quantify the transfection frequency in DNA experiments. Because the *gfp* gene and Cas/gRNA gene cassettes are on the same DNA plasmid, the fluorescent cells can be used as a reasonable proxy for measuring the transfection frequency of CRISPR reagents. On the other hand, co-delivery of GFP and RNP complex is unlikely to achieve the same effect. GFP protein does not have same physical and chemical properties, such as size, charge, and conformation, as the CRISPR-Cas RNPs. In fact, different RNP complexes will have different molecular properties. One strategy to overcome this challenge is to use high quantity of RNP to achieve saturated transfection. Our data (Figure 1B) indicated that no significant difference was observed between different RNP quantities ranged from 10 to 100 µg for both SpCas9 WT and AsCas12a WT. This result suggested that RNP transfection could reach the saturated level when the RNP quantity was above 10 µg. Because 20 µg RNPs were used in all the subsequent protoplast transfection, the comparisons were performed in the saturated transfection manner. Our results demonstrated that all three RNP systems could induce efficient targeted mutations in the protoplasts of diverse plant species. Depending on the targeted sites and species, the mutation frequencies ranged from 2.0% to up to 70.2% that are comparable or higher than the previous studies (Zhang et al., 2021b). Among the tested CRISPR-Cas systems, Cas12a proteins were found to be particularly efficacious in the species evaluated. Of note, LbCas12a RNPs often exhibited higher efficiencies, making them a promising system to achieve genome editing in plants at comparable frequencies to plasmid-based methods.

Another important feature affecting the choice of CRISPR-Cas reagents for plant genome editing is their temperature sensitivity. When delivered as plasmids in plants, both Cas9 and Cas12a demonstrated temperature-dependent nuclease activities (LeBlanc et al., 2018; Malzahn et al., 2019; Schindele and Puchta, 2020; Zhang et al., 2021b). For example, in Arabidopsis and rice somatic cells, CRISPR/Cas9 delivered as plasmids showed 2-5 fold increases in the mutation frequencies at 32°C–37°C compared to those at 22°C (LeBlanc et al., 2018; Malzahn et al., 2019). In addition, Cas12a appeared to be more temperature sensitive. In previous studies, little or no mutagenesis was observed for both AsCas12a WT and LbCas12a WT at 22°C in transformed calli or plants, and required a temperature above 28°C for detectable activity (Malzahn et al., 2019; Banakar et al., 2020; Schindele and Puchta, 2020). In this study, we systematically compared the temperature sensitivity of six Cas9 and Cas12a RNPs under three different temperatures, 22°, 26° and 37°C. In contrast to the previous studies, all Cas9 and Cas12a delivered as RNPs worked efficiently at 22°C. We only observed modest but not statistically significant temperature sensitivity for the tested CRISPR/Cas RNPs. These findings suggested that the CRISPR-Cas systems delivered as RNPs could be more suitable than plasmid-based approaches to induce efficient genome editing under preferred plant tissue culture temperatures.

Specificity is another key consideration in choosing the proper CRISPR/Cas systems. While the LbCas12a V4 RNPs often outperformed AsCas12a Ultra with higher mutation rates, our study demonstrated that AsCas12a Ultra could possess greater sequence specificity in plants. When different CRISPR/Cas RNPs were evaluated to induce mutations in the regenerated calli and plants, we observed the calli transformed with the AsCas12a Ultra RNP exhibited the highest mutation rates (85.2%) followed by those with Cas9 WT and Cas9 HiFi RNPs (55.8 and 34%, respectively). On the contrary, the group transformed with the LbCas12a V4 RNP had very few calli being regenerated. One explanation for this observation could be that the low sequence specificity of LbCas12a V4 may hamper protoplast regeneration. Alternatively, the low regeneration rate observed here could be attributed to the specific gRNA used in this study. Because limited target sites were evaluated in this study, it is possible that location of target sequences on the genome could also impact both efficiency and specificity. Further investigation will be required to fully address this question. Nevertheless, additional improvement can be done to further enhance the performance of the LbCas12a via protein engineering or guide RNA modifications (Ha et al., 2020; Schindele and Puchta, 2020; Zhang et al., 2021b).

It was worth noting that the mutagenesis frequencies achieved in protoplast transfection did not appeared to correlate with the mutation rates achieved in the regenerated calli. In this study, the mutagenesis frequencies in the GFP site ranged from 9.2 to 15.3% for SpCas9 WT, SpCas9 HiFi and AsCas12a Ultra, respectively, in *N. benthamiana* protoplasts. Yet the average mutagenesis frequencies were 55.8, 34 and 85.2% for their transformed calli even without selection. Similar observation was also reported by Park et al. (2019) that the higher editing frequency was observed in *Brassica oleracea* plants regenerated via protoplast mediated RNP delivery. While the mutagenesis frequency was obtained at 2% in protoplasts, they observed a 4-time higher mutagenesis rate (7.8%) in the regenerants. One possible explanation is that RNP molecules could still be present during protoplast regeneration for a period of time and continue to introduce mutations in targeted sites. A time course analysis would be required to further test this hypothesis.

Lastly, our research work demonstrated that the protoplast-based RNP delivery approach can be readily extended to other dicot and monocot plants, such as pennycress, soybean and *Setaria*. Cas12a RNPs, especially LbCas12a V4, generally outperformed the Cas9 RNPs in these plants, consistent with our observations in *N. benthamiana*. Notably, among all the species tested thus far, *S. viridis* exhibited the highest levels of mutagenesis frequencies with all three types of CRISPR-Cas RNPs (58.4–70.2%). Additionally, when the mutational profiles were analyzed from each NGS dataset, the mutation patterns of the Cas9 and Cas12a RNPs were consistent with those obtained from the plasmid delivery method, i.e. less than 5 bp found in the majority indels of Cas9 RNPs and 5 to 20 bp found in the majority indels from Cas12a RNPs (Tang et al., 2017; Weiss et al., 2020). Because protoplasts can be isolated and transformed potentially in a high throughput manner, this highly efficient RNP delivery and genome editing system offers opportunities for large scale genome editing applications, such as CRISPR screening, mutant library construction, and new CRISPR-Cas system screening and improvement (Zhang et al., 2021b). Recently, the plasmid-mediated protoplast transfection systems were used to validate and optimize efficiencies of newly developed base editing and prime editing systems in plant species (Jin et al., 2020; Molla et al., 2020; Tang et al., 2020). The RNP delivery system developed in this study can be leveraged to enable rapid improvement of these new genome editing technologies with less plasmid construction time.

In conclusion, by systematically comparing the CRISPR-Cas systems, our study sheds the light on the choice of RNP reagents for efficient plant genome editing. When delivered as RNP, Cas12a often time outperforms Cas9 to achieve higher genome editing efficacy in multiple plant species. Moreover, protoplast-based RNP delivery does not appear to be overly sensitive to incubation temperatures as plasmid-based methods (Malzahn et al., 2019; Schindele and Puchta, 2020). Thus, the CRISPR-Cas RNP delivery method could be a better choice than plasmid-based approaches for plant genome editing when the preferred plant tissue culture temperature conditions are required. Although the protoplast regeneration process is usually genotype dependent and time consuming, many important vegetable and crop species, such as potato, tomato, lettuce and alfalfa, can be regenerated through protoplasts (Niedz et al., 1985; Monteiro et al., 2003; Woo et al., 2015; Clasen et al., 2016). Additionally, CRISPR-Cas RNPs can be delivered through the biolistic approach in the species that were not amenable for protoplast regeneration, such as wheat and maize (Liang et al., 2017; Dong et al., 2021). Together, our work demonstrated that RNP-mediated protoplast genome editing is a promising technique for rapidly screening and optimizing the CRISPR-Cas systems as well as an effective platform for transgene-free gene edited plants.

## Data Availability

The datasets presented in this study can be found in online repositories. The names of the repository/repositories and accession number(s) can be found below: https://www.ncbi.nlm.nih.gov/bioproject/PRJNA745954.

## References

[B1] Andersson M. Turesson H. Olsson N. Fält A.-S. Ohlsson P. Gonzalez M. N. (2018). Genome Editing in Potato via CRISPR-Cas9 Ribonucleoprotein Delivery. Physiol. Plantarum 164, 378–384. 10.1111/ppl.12731 29572864

[B2] Atkins P. A. Voytas D. F. (2020). Overcoming Bottlenecks in Plant Gene Editing. Curr. Opin. Plant Biol. 54, 79–84. 10.1016/j.pbi.2020.01.002 32143167

[B3] Banakar R. Eggenberger A. L. Lee K. Wright D. A. Murugan K. Zarecor S. (2019). High-frequency Random DNA Insertions upon Co-delivery of CRISPR-Cas9 Ribonucleoprotein and Selectable Marker Plasmid in rice. Sci. Rep. 9, 19902. 10.1038/s41598-019-55681-y 31882637 PMC6934568

[B4] Banakar R. Schubert M. Collingwood M. Vakulskas C. Eggenberger A. L. Wang K. (2020). Comparison of CRISPR-Cas9/Cas12a Ribonucleoprotein Complexes for Genome Editing Efficiency in the rice Phytoene Desaturase (OsPDS) Gene. Rice 13, 4. 10.1186/s12284-019-0365-z 31965382 PMC6973557

[B5] Chen K. Wang Y. Zhang R. Zhang H. Gao C. (2019). CRISPR/Cas Genome Editing and Precision Plant Breeding in Agriculture. Annu. Rev. Plant Biol. 70, 667–697. 10.1146/annurev-arplant-050718-100049 30835493

[B6] Clasen B. M. Stoddard T. J. Luo S. Demorest Z. L. Li J. Cedrone F. (2016). Improving Cold Storage and Processing Traits in Potato through Targeted Gene Knockout. Plant Biotechnol. J. 14, 169–176. 10.1111/pbi.12370 25846201 PMC11389148

[B7] Dong O. X. Yu S. Jain R. Zhang N. Duong P. Q. Butler C. (2020). Marker-free Carotenoid-Enriched rice Generated through Targeted Gene Insertion Using CRISPR-Cas9. Nat. Commun. 11, 1178. 10.1038/s41467-020-14981-y 32132530 PMC7055238

[B8] Dong S. Qin Y. L. Vakulskas C. A. Collingwood M. A. Marand M. Rigoulot S. (2021). Efficient Targeted Mutagenesis Mediated by CRISPR-Cas12a Ribonucleoprotein Complexes in Maize. Front. Genome Ed. 3. 10.3389/fgeed.2021.670529 PMC8525364 34713259

[B9] Fagard M. Vaucheret H. (2000). (Trans)Gene Silencing in Plants: How Many Mechanisms? Annu. Rev. Plant Physiol. Plant Mol. Biol. 51, 167–194. 10.1146/annurev.arplant.51.1.167 15012190

[B10] Garneau J. E. Dupuis M.-È. Villion M. Romero D. A. Barrangou R. Boyaval P. (2010). The CRISPR/Cas Bacterial Immune System Cleaves Bacteriophage and Plasmid DNA. Nature 468, 67–71. 10.1038/nature09523 21048762

[B11] Ha D.-I. Lee J. M. Lee N.-E. Kim D. Ko J.-H. Kim Y.-S. (2020). Highly Efficient and Safe Genome Editing by CRISPR-Cas12a Using CRISPR RNA with a Ribosyl-2′-O-Methylated Uridinylate-Rich 3′-overhang in Mouse Zygotes. Exp. Mol. Med. 52, 1823–1830. 10.1038/s12276-020-00521-7 33162553 PMC8080787

[B12] Horvath P. Barrangou R. (2010). CRISPR/Cas, the Immune System of Bacteria and Archaea. Science 327, 167–170. 10.1126/science.1179555 20056882

[B13] Jin S. Fei H. Zhu Z. Luo Y. Liu J. Gao S. (2020). Rationally Designed APOBEC3B Cytosine Base Editors with Improved Specificity. Mol. Cel 79, 728–740. 10.1016/j.molcel.2020.07.005 32721385

[B14] Jordan N. R. Dorn K. M. Smith T. M. Wolf K. E. Ewing P. M. Fernandez A. L. (2017). A Cooperative Governance Network for Crop Genome Editing. EMBO Rep. 18, 1683–1687. 10.15252/embr.201744394 28928139 PMC5623857

[B15] Jupe F. Rivkin A. C. Michael T. P. Zander M. Motley S. T. Sandoval J. P. (2019). The Complex Architecture and Epigenomic Impact of Plant T-DNA Insertions. Plos Genet. 15, e1007819. 10.1371/journal.pgen.1007819 30657772 PMC6338467

[B16] Kim H. Kim S.-T. Ryu J. Kang B.-C. Kim J.-S. Kim S.-G. (2017). CRISPR/Cpf1-mediated DNA-free Plant Genome Editing. Nat. Commun. 8, 14406. 10.1038/ncomms14406 28205546 PMC5316869

[B17] Kleinstiver B. P. Tsai S. Q. Prew M. S. Nguyen N. T. Welch M. M. Lopez J. M. (2016). Genome-wide Specificities of CRISPR-Cas Cpf1 Nucleases in Human Cells. Nat. Biotechnol. 34, 869–874. 10.1038/nbt.3620 27347757 PMC4980201

[B18] Kurgan G. Turk R. Li H. Roberts N. Rettig G. R. Jacobi A. M. (2021). CRISPAltRations: a Validated Cloud-Based Approach for Interrogation of Double-Strand Break Repair Mediated by CRISPR Genome Editing. Mol. Ther. - Methods Clin. Dev. 21, 478–491. 10.1016/j.omtm.2021.03.024 33981780 PMC8082044

[B19] LeBlanc C. Zhang F. Mendez J. Lozano Y. Chatpar K. Irish V. F. (2018). Increased Efficiency of Targeted Mutagenesis by CRISPR/Cas9 in Plants Using Heat Stress. Plant J. 93, 377–386. 10.1111/tpj.13782 29161464

[B20] Li J. Stoddard T. J. Demorest Z. L. Lavoie P.-O. Luo S. Clasen B. M. (2016). Multiplexed, Targeted Gene Editing inNicotiana Benthamianafor Glyco-Engineering and Monoclonal Antibody Production. Plant Biotechnol. J. 14, 533–542. 10.1111/pbi.12403 26011187 PMC11389102

[B21] Liang Z. Chen K. Li T. Zhang Y. Wang Y. Zhao Q. (2017). Efficient DNA-free Genome Editing of Bread Wheat Using CRISPR/Cas9 Ribonucleoprotein Complexes. Nat. Commun. 8, 14261. 10.1038/ncomms14261 28098143 PMC5253684

[B22] Liu J. Nannas N. J. Fu F.-f. Shi J. Aspinwall B. Parrott W. A. (2019). Genome-scale Sequence Disruption Following Biolistic Transformation in rice and maize. Plant Cell 31, 368–383. 10.1105/tpc.18.00613 30651345 PMC6447018

[B23] Malnoy M. Viola R. Jung M.-H. Koo O.-J. Kim S. Kim J.-S. (2016). DNA-free Genetically Edited grapevine and Apple Protoplast Using CRISPR/Cas9 Ribonucleoproteins. Front. Plant Sci. 7, 1904. 10.3389/fpls.2016.01904 28066464 PMC5170842

[B24] Malzahn A. A. Tang X. Lee K. Ren Q. Sretenovic S. Zhang Y. (2019). Application of CRISPR-Cas12a Temperature Sensitivity for Improved Genome Editing in rice, maize, and Arabidopsis. BMC Biol. 17, 9. 10.1186/s12915-019-0629-5 30704461 PMC6357469

[B25] Menz J. Modrzejewski D. Hartung F. Wilhelm R. Sprink T. (2020). Genome Edited Crops Touch the Market: A View on the Global Development and Regulatory Environment. Front. Plant Sci. 11, 586027. 10.3389/fpls.2020.586027 33163013 PMC7581933

[B26] Molla K. A. Shih J. Yang Y. (2020). Single-nucleotide Editing for Zebra3 and Wsl5 Phenotypes in rice Using CRISPR/Cas9-mediated Adenine Base Editors. aBIOTECH 1, 106–118. 10.1007/s42994-020-00018-x PMC9590491 36304716

[B27] Monteiro M. Appezzato-da-Glória B. Valarini M. J. Oliveira C. A. d. Vieira M. L. C. (2003). Plant Regeneration from Proroplasts of Alfalfa (Medicago Sativa) via Somatic Embryogenesis. Sci. Agric. (Piracicaba, Braz. 60, 683–689. 10.1590/s0103-90162003000400012

[B28] Niedz R. P. Rutter S. M. Handley L. W. Sink K. C. (1985). Plant Regeneration from Leaf Protoplasts of Six Tomato Cultivars. Plant Sci. 39, 199–204. 10.1016/0168-9452(85)90175-x

[B29] Park S.-C. Park S. Jeong Y. J. Lee S. B. Pyun J. W. Kim S. (2019). DNA-free Mutagenesis of GIGANTEA in *Brassica oleracea* Var. Capitata Using CRISPR/Cas9 Ribonucleoprotein Complexes. Plant Biotechnol. Rep. 13, 483–489. 10.1007/s11816-019-00585-6

[B30] Ruiz M. T. Voinnet O. Baulcombe D. C. (1998). Initiation and Maintenance of Virus-Induced Gene Silencing. Plant Cell 10, 937–946. 10.1105/tpc.10.6.937 9634582 PMC144041

[B31] Schindele P. Puchta H. (2020). Engineering CRISPR/Lb Cas12a for Highly Efficient, Temperature‐tolerant Plant Gene Editing. Plant Biotechnol. J. 18, 1118–1120. 10.1111/pbi.13275 31606929 PMC7152607

[B32] Subburaj S. Chung S. J. Lee C. Ryu S.-M. Kim D. H. Kim J.-S. (2016). Site-directed Mutagenesis in Petunia × Hybrida Protoplast System Using Direct Delivery of Purified Recombinant Cas9 Ribonucleoproteins. Plant Cel Rep 35, 1535–1544. 10.1007/s00299-016-1937-7 26825596

[B33] Svitashev S. Schwartz C. Lenderts B. Young J. K. Mark Cigan A. (2016). Genome Editing in maize Directed by CRISPR-Cas9 Ribonucleoprotein Complexes. Nat. Commun. 7, 13274. 10.1038/ncomms13274 27848933 PMC5116081

[B34] Tang X. Lowder L. G. Zhang T. Malzahn A. A. Zheng X. (2017). A CRISPR–Cpf1 System for Efficient Genome Editing and Transcriptional Repression in Plants. Nat. plants 3, 17103. 10.1038/nplants.2017.103 28628131

[B35] Tang X. Sretenovic S. Ren Q. Jia X. Li M. Fan T. (2020). Plant Prime Editors Enable Precise Gene Editing in rice Cells. Mol. Plant 13, 667–670. 10.1016/j.molp.2020.03.010 32222487

[B36] Vakulskas C. A. Dever D. P. Rettig G. R. Turk R. Jacobi A. M. Collingwood M. A. (2018). A High-Fidelity Cas9 Mutant Delivered as a Ribonucleoprotein Complex Enables Efficient Gene Editing in Human Hematopoietic Stem and Progenitor Cells. Nat. Med. 24, 1216–1224. 10.1038/s41591-018-0137-0 30082871 PMC6107069

[B37] Weiss T. Wang C. Kang X. Zhao H. Elena Gamo M. Starker C. G. (2020). Optimization of Multiplexed CRISPR/Cas9 System for Highly Efficient Genome Editing in Setaria Viridis. Plant J. 104, 828–838. 10.1111/tpj.14949 32786122

[B38] Woo J. W. Kim J. Kwon S. I. Corvalán C. Cho S. W. Kim H. (2015). DNA-free Genome Editing in Plants with Preassembled CRISPR-Cas9 Ribonucleoproteins. Nat. Biotechnol. 33, 1162–1164. 10.1038/nbt.3389 26479191

[B39] Zhang L. Zuris J. A. Viswanathan R. Edelstein J. N. Turk R. Thommandru B. (2021a). AsCas12a Ultra Nuclease Facilitates the Rapid Generation of Therapeutic Cell Medicines. Nat. Commun. 12, 3908. 10.1038/s41467-021-24017-8 34162850 PMC8222333

[B40] Zhang Y. Zhang F. Li X. Baller J. A. Qi Y. Starker C. G. (2013). Transcription Activator-like Effector Nucleases Enable Efficient Plant Genome Engineering. Plant Physiol. 161, 20–27. 10.1104/pp.112.205179 23124327 PMC3532252

[B41] Zhang Y. Iaffaldano B. Qi Y. (2021b). CRISPR Ribonucleoprotein-Mediated Genetic Engineering in Plants. Plant Commun. 2, 100168. 10.1016/j.xplc.2021.100168 33898980 PMC8060726

[B42] Zhang Y. Liang Z. Zong Y. Wang Y. Liu J. Chen K. (2016). Efficient and Transgene-free Genome Editing in Wheat through Transient Expression of CRISPR/Cas9 DNA or RNA. Nat. Commun. 7, 12617. 10.1038/ncomms12617 27558837 PMC5007326

[B43] Zhang Y. Malzahn A. A. Sretenovic S. Qi Y. (2019). The Emerging and Uncultivated Potential of CRISPR Technology in Plant Science. Nat. Plants 5, 778–794. 10.1038/s41477-019-0461-5 31308503

[B44] Zhang Y. Ren Q. Tang X. Liu S. Malzahn A. A. Zhou J. (2021c). Expanding the Scope of Plant Genome Engineering with Cas12a Orthologs and Highly Multiplexable Editing Systems. Nat. Commun. 12, 1944. 10.1038/s41467-021-22330-w 33782402 PMC8007695

