# Supplementary material for: Efficiency, Specificity and Temperature Sensitivity of Cas9 and Cas12a RNPs for DNA-free Genome Editing in Plants
Source: Front Genome Ed. 2022 Jan 12;3:760820. doi: 10.3389/fgeed.2021.760820 (PMC8790294; doi:10.3389/fgeed.2021.760820)
Supplement: Supplementary file 1 [file DataSheet1.docx]

# Supplementary Materials

Efficiency, specificity, and temperature sensitivity of Cas9 and Cas12a RNPs for DNA-free genome editing in plants

Raviraj Banakar^1,2,3^, Mollie Schubert^4^, Gavin Kurgan^4^, Krishan Mohan Rai^1,2,3^, Sarah Beaudoin^4^, Michael A. Collingwood^4^, Christopher A. Vakulskas^4^, Kan Wang^5,6^, Feng Zhang^1,2,3^*

^1^Department of Plant and Microbial Biology, University of Minnesota, St. Paul, Minnesota, USA

^2^Center for Precision Plant Genomics, University of Minnesota, St. Paul, Minnesota, USA

^3^Center for Genome Engineering, University of Minnesota, St. Paul, Minnesota, USA

^4^Integrated DNA Technologies, Coralville, Iowa, USA

^5^Department of Agronomy, Iowa State University, Ames, Iowa, USA

^6^Crop Bioengineering Center, Iowa State University, Ames, Iowa, USA

*****Corresponding Author,

zhangumn@umn.edu

**Figure S1. The workflow of the CRISPR/Cas RNP-mediated gene editing in protoplasts.**


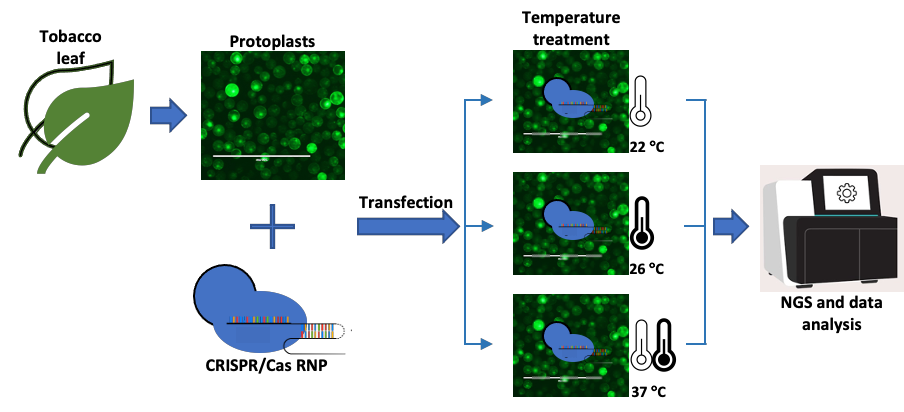


**Figure S2. Sequence alignment of the *NbPDS1* and *PDS2* coding regions**

The Cas12a targeted sites were highlighted in yellow with the TTTG PAM sequences underlined. SNPs in the flanking regions of the targeted sites were used to discriminate the *PDS1* sequence from *PDS2*.

**Figure S3. Plant materials and protoplasts from Pennycress, *Setaria viridis* and soybean**

**
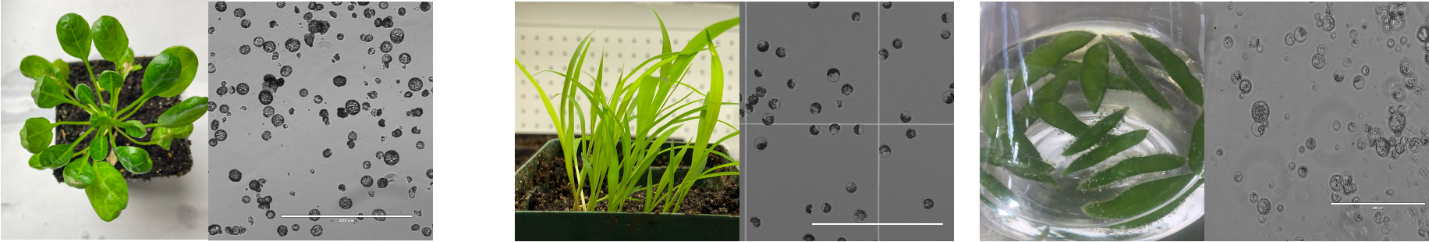
**

Pennycress

*Setaria viridis*

Soybean

The plant materials were shown for protoplast isolation and transfection from pennycress (2-week old plants), *Setaria viridis* (2-week old plants) and soybean (R2 stage seed pods; 14 days after fertilization). The protoplasts were shown before RNP transfection with the white scale bar indicating 400 µm.

**Table S1. Summary of the CRISPR-Cas target sites.**

| **Species** | **Nuclease types** | **I.D.** | **Spacer** | **PAM** | **Targeted gene** |
| --- | --- | --- | --- | --- | --- |
| *N. benthamiana* | SpCas9 | gRNA 1_GFP | CTTGTCACTACTTTCTCTTA | TGG | GFP |
| *N. benthamiana* | Cas12a | gRNA 1_GFP | TCTTATGGTGTTCAATGCTTT | TTTC | GFP |
| *N. benthamiana* | SpCas9 | gRNA 2_GFP | AGTAGTGCAAATAAATTTAA | GGG | GFP |
| *N. benthamiana* | Cas12a | gRNA 2_GFP | CAGTAGTGCAAATAAATTTAA | TTTC | GFP |
| *N. benthamiana* | Cas12a | PDS1 | GAGCTCGAGGTCTTCGTTGGG | TTTG | PDS1 |
| *Thlaspi arvense* | SpCas9 | gRNA1 | TGGCTCTCTACATCGTAACC | CGG | FAE1 ^1^ |
| *Thlaspi arvense* | Cas12a | gRNA1 | GCTCTCTACATCGTAACCCG | TTTG | FAE1 |
| *Setaria viridis* | SpCas9 | gRNA1_Drm1 | GGTATGGGTTGGTCTCAACA | AGG | DRM1^2^ |
| *Setaria viridis* | Cas12a | gRNA1_Drm1 | GTATGGGTTGGTCTCAACAAG | TTTG | DRM1 |
| *Glycine max* | SpCas9 | gRNA1_Fad2-1A | TTTTAGTCCCTTATTTCTCA | TGG | FAD2-1A |
| *Glycine max* | Cas12a | gRNA1_Fad2-1A | GTCCCTTATTTCTCATGGAA | TTTA | FAD2-1A^3^ |

The superscript number indicated the gRNA sequences that were tested in the previous studies.

1. Jarvis, Brice A., Trevor B. Romsdahl, Michaela G. McGinn, Tara J. Nazarenus, Edgar B. Cahoon, Kent D. Chapman, and John C. Sedbrook. 2021. “CRISPR/Cas9-Induced Fad2 and Rod1 Mutations Stacked With Fae1 Confer High Oleic Acid Seed Oil in Pennycress (Thlaspi Arvense L.).” *Frontiers in Plant Science* 12 (April): 652319.
2. Weiss, Trevor, Chunfang Wang, Xiaojun Kang, Hui Zhao, Maria Elena Gamo, Colby G. Starker, Peter A. Crisp, et al. 2020. “Optimization of Multiplexed CRISPR/Cas9 System for Highly Efficient Genome Editing in Setaria Viridis.” *The Plant Journal: For Cell and Molecular Biology*, August. https://doi.org/10.1111/tpj.14949.
3. Kim, Hyeran, and Jisun Choi. 2020. “A Robust and Practical CRISPR/CrRNA Screening System for Soybean Cultivar Editing Using LbCpf1 Ribonucleoproteins.” *Plant Cell Reports*, September. https://doi.org/10.1007/s00299-020-02597-x.

**Table S2. Summary of the primer sequences.**

| **Species** | **Target loci** | **Primer names** | **Primer sequences** |
| --- | --- | --- | --- |
| *N. Benthamiana* | mGFP | GFP Forward | acactctttccctacacgacgctcttccgatctAGATGGTGATGTTAATGGGTACA |
| *N. Benthamiana* | mGFP | GFP Reverse | gtgactggagttcagacgtgtgctcttccgatctCCTTGAAGAAGATGGTCCTCTC |
| *N. Benthamiana* | PDS | PDS Forward | acactctttccctacacgacgctcttccgatctGGACTTGTTTCTGCCGTTAATTT |
| *N. Benthamiana* | PDS | PDS Reverse | gtgactggagttcagacgtgtgctcttccgatctCCATCATCACACTTTCGCATTC |
| *Thlaspi arvense* | FAE1 | FAE1 Forward | acactctttccctacacgacgctcttccgatctCACAAACGATCTTCACCACTTC |
| *Thlaspi arvense* | FAE1 | FAE1 Reverse | gtgactggagttcagacgtgtgctcttccgatctCCGTAAAGGATCGGCTAATCT |
| *Setaria viridis* | Drm1a | Drm1a Forward | acactctttccctacacgacgctcttccgatctTGGCCCTAACAAACAGTGAA |
| *Setaria viridis* | Drm1a | Drm1a Reverse | gtgactggagttcagacgtgtgctcttccgatctTCGTGTGATCCTTTGGGAAG |
| *Glycine max* | Fad2-1A | Fad2-1A Forward | acactctttccctacacgacgctcttccgatctCACCATGCCTTCAGCAAGTA |
| *Glycine max* | Fad2-1A | Fad2-1A Reverse | gtgactggagttcagacgtgtgctcttccgatctTTCCTAGAGGGTTGTTCAGGTA |

Note: in the primer sequences, the lowercase bases are the universal tails for the NGS barcoding PCR, while the uppercase bases are the target specific sequences.

**Table S3. Summary of mutation genotypes identified from E1 mutant *N. benthamiana* plants.**

| **E0 Plant ID** | **Cas RNP transfected** | **E1 Plant ID** | **mutation genotypes^1^** | **GFP phenotypes** |
| --- | --- | --- | --- | --- |
| PT301 | SpCas9 WT | 1 | +1(T) / +1(T) | GFP negative |
|  |  | 2 | +1(T) / WT | Semi GFP |
|  |  | 3 | +1(T) / +1(T) | GFP negative |
|  |  | 4 | +1(T) / +1(T) | GFP negative |
|  |  | 5 | +1(T) / +1(T) | GFP negative |
| PT304 | SpCas9 WT | 1 | +1(T) / +1(A) | GFP negative |
|  |  | 2 | +1(A) / +1(A) | GFP negative |
|  |  | 3 | +1(T) / +1(A) | GFP negative |
|  |  | 4 | +1(T) / +1(T) | GFP negative |
|  |  | 5 | +1(T) / +1(A) | GFP negative |
| PT320 | AsCas12a_Ultra | 1 | -6 (AATGCT) / -10 (TTCAATGCTT) | GFP negative |
|  |  | 2 | -5 (CAATG)/ -10 (TTCAATGCTT) | GFP negative |
|  |  | 3 | -5 (CAATG)/ -6 (AATGCT) | GFP negative |
|  |  | 4 | -5 (CAATG)/ -10 (TTCAATGCTT) | GFP negative |
|  |  | 5 | -6 (AATGCT) / -10 (TTCAATGCTT) | GFP negative |

Note: The size of insertion and deletion mutations were represented by positive or negative number, respectively. The inserted or deleted nucleotides were shown in the paratheses.
